# Confronting CLABSI Disparities: The Role of REaL Variables, Data Transparency, and Intentional Process Measurement in Achieving Equitable Outcomes

**DOI:** 10.1097/pq9.0000000000000606

**Published:** 2022-10-03

**Authors:** Megan D. Stimpson, Shanda M. Johnson, Lara R. Wood, Brendan Bettinger, Paul J. Sharek, Bonnie Fryzlewicz, Danielle M. Zerr

**Affiliations:** From the * Seattle Children’s Hospital; † Department of Pediatrics, University of Washington School of Medicine.

## Background:

Disparities in outcomes continue to exist in healthcare. In 2019, we evaluated non-mucosal barrier injury central line associated blood stream infection (CLABSI) rates by race, ethnicity and language (REaL) variables and found rates were 2.92 per 1,000 central line days for patients identifying as Black or African American and 1.37 per 1,000 central line days for patients that speak a language other than English (LOE), despite an overall non-mucosal barrier injury CLABSI rate of 1.13 per 1000 central line days.

## Objective:

Determine if interventions specifically targeting Black/African American and LOE patient populations improve, or eliminate, disparities in non-MBI CLABSI rates at a free standing academic quaternary children’s hospital.

## Methods:

**Figure 1.**
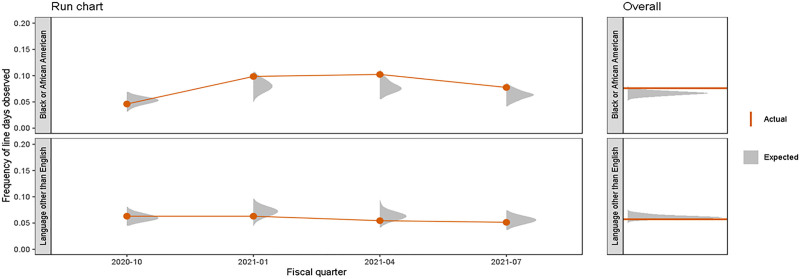
Maintenance observation ratio.

Black/African American and LOE CLABSI reduction efforts were led by a multidisciplinary oversight team and sponsored by senior executives. The team met bi-weekly, and oversaw weekly huddles where process (compliance with maintenance bundle) and outcome data were presented by REaL variables for transparency and learning. Individual hospital units also received their CLABSI maintenance bundle and outcome data stratified by REaL. In addition, a ‘maintenance observation ratio’ metric was created by hospital unit to illustrate whether the frequency of central line observation audits occurred at a rate proportionate to the number of expected audits given the number of line days a given patient sub-population contributed to the whole (Fig. 1). Once it was apparent that disparities existed in the non-MBI CLABSI rates and the observation ratios, the oversight team developed separate Key Driver Diagrams for Black/African American patients and LOE patients to identify specific interventions to eliminate the disparities. Projects ranged from gathering feedback from families that identified as Black/African American and families who speak a language other than English, to revising policies, procedures, and patient and family educational materials to be more inclusive and equitable.

## Results:

**Figure 2.**
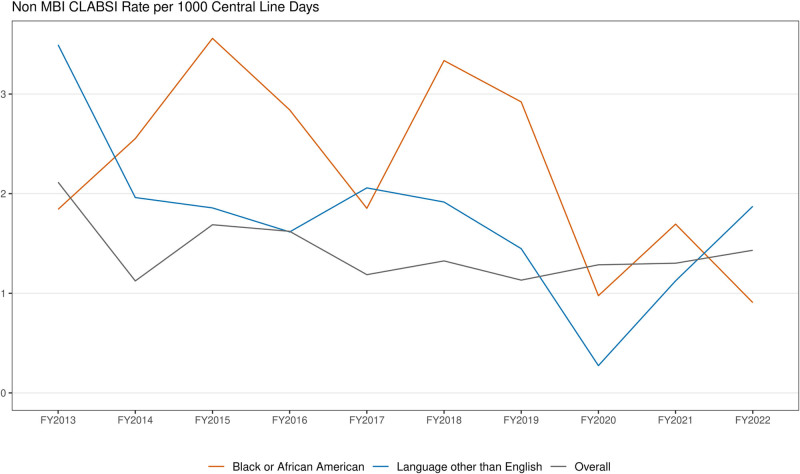
Non-MBI CLABSI rate per 1000 central line days by REaL variables

Non-MBI CLABSI rates decreased over time in all groups and disparities were lessened in the Black/African American population (Fig. 2).

## Conclusions:

Evaluation of process (maintenance bundle compliance) and outcome measures by REaL variables is a critical step in eliminating healthcare disparities. Embedding race and language-based strata into CLABSI measures, and making these data visible, can expose disparities and inform highest priority improvement efforts. It is essential to share these data transparently at all levels of the organization. Individual Key Driver Diagrams based on race and language strata are useful tools to organize interventions and assist teams in maintaining focus on the goal.

## DISCLOSURE

The authors have no financial interest to declare in relation to the content of this article.

